# Imaging tumour response - challenges

**DOI:** 10.1186/1470-7330-15-S1-O14

**Published:** 2015-10-02

**Authors:** Finn Rasmussen

**Affiliations:** 1 Department of Radiology, Aarhus University Hospital, Denmark

## 

RECIST criteria use changes in tumour diameters to quantitatively obtain response assessments for traditional chemotherapeutic cancer treatments: Positive responses are identified by reductions in tumour size while tumour growth signifies non-response.

Traditional chemotherapies work primarily by damaging cells that are undergoing rapid growth and division. Immunotherapies work by stimulating the immune system to reject and destroy tumour cells. Targeted cancer therapies block the growth of cancer cells by interfering with specific molecules needed for tumour growth and carcinogenesis. These oncological treatments as well as stereotactic radiation therapy and local treatments, such as image-guided ablations and **t**rans**a**rterial **c**hemo **e**mbolization (TACE) can be used alone or in combination. Local treatment, radiation therapy, immunotherapy and targeted therapy can each result in an increased tumour diameter in spite of treatment response at the cellular level and increased survival times. As a result, tumour diameter can no longer stand alone when assessing oncological treatment strategies

Tumour heterogeneity and contrast enhancement often change during treatment, and tumour enhancement combined with tumour size has been shown to be successful in assessing treatment response in gastrointestinal stromal tumours (CHOI criteria). Modified CHOI criteria could potentially be used for monitoring the effect of oncological treatment strategies in metastatic renal cell cancer and hepatocellular carcinoma.

Dynamic contrast-enhanced imaging (DCE-US, DCE-MRI and DCE-CT) uses changes in tumour enhancement following the intravenous injection of a contrast media to create a time-versus-signal curve. From such curves, reliable quantitative estimates of blood flow, blood volume and permeability can be obtained. Diffusion-weighted MRI and PET can also be used to obtain quantitative measures of physiological processes. A major advantage of DWI-MRI and PET is the possibility of whole body coverage, while DCE imaging is limited to a single tumour or a comparatively small region.

Although quantitative assessments of oncological treatment response using physiological parameters have been shown to be superior to size-based response in several studies, the methods mentioned are not yet standardized, and randomized multicentre studies have not yet been performed. When planning such studies, it is important to note that clinical endpoints such as progression free survival and overall survival are superior to morphological imaging results.

In the routine assessment of oncological treatment responses, it is becoming increasingly important for the radiologists to know exactly what kind(s) of treatment that the patient has received. Simply reporting changes in tumour size using RECIST is no longer sufficient considering the large percentage of patients who receive advanced and often combined treatment regimens.

